# The organization of ideological discourse in times of unexpected crisis: Explaining how COVID-19 is exploited by populist leaders

**DOI:** 10.1177/1742715020926783

**Published:** 2020-05-12

**Authors:** Ajnesh Prasad

**Affiliations:** Tecnologico de Monterrey, Mexico; Royal Roads University, Canada

**Keywords:** COVID-19, crisis, ideological discourse, India, populism

## Abstract

Using the persecution of Muslims in India that is currently taking place against the backdrop of the COVID-19 global pandemic as an illustrative case, this essay identifies the dynamics of *the* *organization of ideological discourse* by populist leaders in times of unexpected crisis. The organization of ideological discourse represents strategic, discursive acts committed by populist leaders aimed at foregrounding social conditions that would function in the advancement of various political ends—whether those ends may be the consolidation of power, the undermining of institutional systems of checks and balances, the implementation of exclusionary or injurious policies against disenfranchised constituents, the suspension of civil liberties, or a combination thereof. It is engendered through a three-stage process. In the first stage, surface-level validation by legitimate institutional actors confirms preconceived ideas about a constructed enemy. In the second stage, inflammatory rhetoric is deployed by populist leaders, which scapegoat that constructed enemy. These two stages culminate to create widespread moral panic in society. With moral panic firmly established, in the third stage an environment of fear and paranoia becomes susceptible to the enactment of symbolic and physical violence against the constructed enemy. The essay concludes with some words on the pressing need to deconstruct ideologically motivated discourses related to COVID-19.

“Those who cannot remember the past are condemned to repeat it.”

– George Santayana

## From 1938 Germany to 2020 India

On 7 November 1938, German diplomat Ernst vom Roth, while stationed in Paris, was shot multiple times by Herschel Grynszpan, a Jewish teenager. vom Roth succumbed to his injuries two days later. The events that unfolded in Nazi Germany in the immediate aftermath of vom Roth’s death has, perhaps, never been more timely to revisit than today. The events would come to be popularized as Kristallnacht.

Known in English as ‘The Night of the Broken Glass’, Kristallnacht represents the violent acts that transpired in Germany in the late evening of 9 November 1938 and carried over into the early morning hours of the following day. During that night, Jewish owned properties, including synagogues, homes, and businesses were vandalized or seized, dozens of Jews were killed, and countless other Jews were physically assaulted (Steinweis, 2009). While Kristallnacht was ostensibly attributed by Nazi officials and their sympathizers as being (the logical) retaliatory outcome for the assassination of vom Roth, it was in actuality—as the benefit of historical foresight reveals—the pivotal, though unexpected, crisis that Hitler exploited in efforts to materially advance the antisemitic ideology that foregrounded Nazism. That is, Hitler used vom Roth’s assassination to expedite Nazism’s ultimate ideological objective: The Final Solution.

Kristallnacht vividly illuminates how the politicization of an unexpected crisis can be mobilized by populist leaders in the service of their underlying ideological agenda. Over eight decades later, such is occurring with the global pandemic of COVID-19. Namely, while worldwide attention has been, almost wholly, focused on immediate concerns on how best to contain the spread of the virus, unscrupulous populist leaders are invoking COVID-19 with ulterior, often malicious, motives. While this phenomenon is manifesting all over the world, perhaps nowhere else is it more evident than in India. Indeed, populist leaders of the reigning Bharatiya Janata Party (BJP) have strategically crafted a set of discourses, shrouded in the veneer of COVID-19 concerns, to promote the party’s longstanding Hindu nationalist—and, concomitantly, anti-Muslim—ideology of Hindutva. In this essay, I label the process through which an unexpected crisis is exploited by populist leaders, *the* *organization of ideological discourse*.

The idea of the organization of ideological discourse is informed by readings of hegemony from the works of Antonio Gramsci (1971) and ideology from the works of Slavoj Zizek (1989). Put simply, the organization of ideological discourse represents strategic, discursive acts committed by populist leaders aimed at foregrounding social conditions that would function in advancing their political ends. These ends may be the consolidation of power, the undermining of institutional systems of checks and balances, the implementation of exclusionary or injurious policies against disenfranchised constituents, the suspension of civil liberties, or a combination thereof.

In the remainder of this essay, I have two aims. My first aim is to offer some empirical substance to how I am conceiving of the organization of ideological discourse in times of unexpected crisis. On this point, I outline how the BJP has propagated discourses related to COVID-19 with a particular ideological intent—to conjure moral panic among Hindus and, thereby, establish an environment of fear and paranoia necessary to encourage the enactment of symbolic and physical violence against Muslims in India. My second aim is to offer some cautionary words moving forward. On this point, I describe the urgency to deconstruct ideologically motivated discourses related to COVID-19 currently being circulated by populist political leaders around the world.

## The politicization of COVID-19 by Hindu nationalists in India

Coming into this year, the reigning BJP, led by Prime Minister Narendra Modi, engaged in concerted efforts to marginalize India’s Muslim population by pursuing its longstanding ideology of Hindutva. Using the most generous of terms, Hindutva is a political ideology for the BJP that “[defines] Indian culture in terms of Hindu values” (Ayyub, 2019). In its more pernicious form, it is a political ideology that is predicated on the belief that “the sacred motherland of India is for Hindus only” (Masood and Nisar, 2020: 163).

Many of the BJP’s efforts to advance Hindutva culminated in 2016, when the Lok Sabha—the country’s lower house of parliament—passed the Citizenship Amendment Bill (CAB). The CAB proposed to make illegal immigrants from Afghanistan, Bangladesh, and Pakistan eligible for Indian citizenship so long as they are not Muslim. While members of the BJP purported that the bill was a progressive move in that it sought to protect minorities who had been subjected to persecution in their home countries, its singular exclusion of Muslims—irrespective of the sectarian-based persecution that Muslims may have experienced in their home countries (e.g., Ahmadiyas in Pakistan)—exposed the bill’s underlying discriminatory project (Bhat, 2019). The tabled bill cleared India’s upper house of parliament, the Rajya Sabha, on 11 December 2019.

At the same time that mass protests were being convened by conscientious citizens across the country to object to the passing of the CAB, COVID-19 spread across the global and, on 11 March 2020, the World Health Organization declared it a global pandemic. India was certainly not immune from the impact of the virus. As confirmed cases of COVID-19 grew exponentially in the country, Modi instituted a draconian-style, countrywide lockdown that took effect on 25 March (Wasdani and Prasad, 2020).

Leaders within the BJP seized on the unexpected crisis engendered by the global pandemic by aligning the discourse over COVID-19 with currents of their Hindutva ideology. This politicization of COVID-19 was orchestrated in a three-stage process that singled out Muslims as the carriers and the spreaders of the virus and, in the process, constructed them as the country’s enemy. The first two stages were liminal inasmuch as they enabled one another—one stage would be less tenable without the enactment of the other. The first two stages, functioning in concert, would activate the third stage, which was the precise juncture at which the ideology of Hindutva materialized into myriad violent acts against the constructed enemy.

The first stage required surface-level validation by legitimate institutional actors to confirm preconceived ideas about the constructed enemy. In this case, it affixed blame for the virus to India’s Muslim population. This was accomplished when rumors began to circulate that COVID-19 was introduced and spread in India by members of the Islamic group, the Tablighi Jamaat. The rumors acquired wide currency among Hindu nationalists as hashtags like #CoronaJihad, #CrushTablighiSpitters, and #TablighiJamatVirus began to trend on Twitter (Gilbert, 2020; Perrigo, 2020). These rumors would become codified when it was given some level of legitimacy by various institutional actors. In this case, institutional legitimacy came when, among other things:

1. On 31 March, the Delhi Police Crime Branch formally lodged a First Information Report against Maulana Saad Kandhlawi, the great grandson of the Tablighi Jamaat’s founder and the current head of the Nizamuddin Marqaz Mosque—the headquarters of the organization. Saad was accused of violating the Epidemic Diseases Act for not observing the government’s instituted social distancing protocols (Bisht and Naqvi, 2020);
2. On 2 April, the Ministry of Home Affairs, blacklisted foreigners who were Tablighi members by prohibiting them from engaging in Jamaat activities (Hussain, 2020; for an overview of activities practiced by Tablighi Jamaat members, see Rauf and Prasad, 2020), and;
3. The Indian government dedicating a specific column in its daily briefings on COVID-19 to the Tablighi Jamaat (Aproovanand, 2020). Lav Agarawal, a secretary in the health ministry, has continuously associated the virus with the Islamic group (Yasir, 2020).

Taken collectively, these acts—each articulated by a legitimate institutional actor—functioned to validate the rumors on the origin of the virus that had been promulgating in the country.

The achievement of surface-level validation provided the necessary grounds to make a liminal move to the second stage: the demonization of Muslims in the country through inflammatory political rhetoric (Li and Prasad, 2018) . In this case, surface-level validation from legitimate institutional actors justified the demonization of Muslims as they came to be viewed as an existential threat to both India and Hinduism. It was in this space where BJP leaders deployed targeted, vitriol attacks on social media and elsewhere. For example, Kamil Mishra, a local BJP leader, tweeted: “Tablighi Jamaat people have begun spitting on the doctors and other health workers. It’s clear, their aim is to infect as many people as possible with coronavirus and kill them” (Ellis-Petersen and Rahman, 2020). In a similar vein, Amit Malviya, the national head of BJP’s information and technology division, tweeted on 1 April:

Delhi’s dark underbelly is exploding! Last 3 months have seen an Islamic insurrection of sorts, first in the name of anti-CAA protests from Shaheen Bagh to Jamia, Jaffrabad to Seelampur. And now the illegal gathering of the radical Tablighi Jamaat at the markaz. It needs a fix! (Pasha, 2020).

On 4 April, Rajeev Bindal, the Himachal Pradesh BJP chief, asserted that, “the Centre and state governments are leaving no stone unturned in the decisive fight against COVID-19 but some people, including Tablighi Jamaat, members are moving like human bombs to thwart their efforts.” This sentiment was echoed days later when, on 9 April, senior BJP leader and former chief minister of Maharashtra, Devendra Fadnavis, invoked a tried and tested Islamophobic trope by likening Tablighi Jamaat members to “human bombs” (Pasha, 2020).

Before proceeding to discuss the third stage, two points merit note: one relates to the general nature of the organization of ideological discourse and one relates to its operation as specifically relevant to this case. On the latter point, the Tablighi Jamaat was an especially convenient scapegoat for the accomplishment of the first two stages as its members conspicuously embody Islam both substantively and symbolically. In terms of substance, the Tablighi Jamaat is a missionary organization established with the primary purpose of having its members travel community to community disseminating the word of Mohammad (Rauf et al., 2019). In terms of symbol, the Tablighi Jamaat ascribes to an orthodox interpretation of Islam and, thus, its members’ grooming habits, consumption patterns, mannerisms, and garb clearly demarcate them from adherents of other religions (Rauf and Prasad, 2020).

On the former point, it must be underscored that the first two stages for the organization of ideological discourse are mutually constituting. The intent of their interaction is the creation of widespread moral panic in society. Moral panic constructs an imaginary enemy (in this case, certain practitioners of Islam), which is presented as a ubiquitous threat to the stability of society as well as to its bona fide citizens (Cohen, 1972). Within the purview of a moral panic, many come to believe that without the eradication of the imaginary enemy, society in its existing form—including its culture, tradition, religion, etc.—would crumble. Hence, moral panic casts urgency upon righteous individuals to defend society from the constructed enemy who seeks to infect it.

Surface-level validation by legitimate institutional actors complemented by incendiary rhetoric from political leaders, paves the way to the third stage: the orchestration of an environment of fear and paranoia that is amenable to the enactment of symbolic and physical violence against the constructed enemy. It is critical to note that at this stage there is an important shift in the discourse. Namely, while the first two stages targeted an easy but definable enemy—members of the Tablighi Jamaat who were linked, however speciously, to the spread of COVID-19—at this stage, the specter of violence was expanded to cover the group’s body politic. To put it another way, it came to encompass all Muslims. Once the pretense of COVID-19 was no longer necessary, Muslims become undifferentiated and the Hindu nationalists’ underlying ideological project of Hindutva would re-emerge more palpably.

Stories of violence experienced by Muslims at the hands of Hindus who associated them with the cause of COVID-19 abound. On 5 April, Mehboob Ali, a 22-year-old Muslim man from the village of Harewali was beaten mercilessly by others in his village. The beating was video recorded and posted on social media. During the beating, his assailants can be heard yelling and demanding answers from him: “Tell us your plan! Was your plan to spread corona?” (Gettleman et al., 2020). On the very same day, Dilshad Muhamud, a 37-year-old Muslim man from a different village hanged himself after villagers accused him of carrying and spreading the virus. Although Muhamud had been tested and confirmed to be negative of the virus hours earlier, the social stigmatization he felt from other villagers led him to suicide. As tragic is the case of Ambreen Khan, a Muslim woman from the state of Punjab. On 10 April, Khan drove home following a work shift at a local hospital. Her vehicle was surrounded by a mob of men. She was forcibly pulled out of her, cursed at, assaulted, and molested. During the abuse, the mob can be heard chanting, “go back to Pakistan” (Yasir, 2020).

These three illustrative cases are all the outcomes of the third stage of the organization of ideological discourse. The effective appropriation of COVID-19 for political purposes generated a moral panic so intense that merely being Muslim qualified someone to being subjected to communal violence. What is perhaps most alarming, and poignant, is the fact that in all of the cases the Hindu assailants were not strangers to the Muslim victims. The beating experienced by Ali, the stigmatization felt by Muhamud, and the physical and sexual assault encountered by Khan, were committed by their respective neighbors—people whom the victims lived alongside for years. This speaks to the insidiousness with which the organization of ideological discourse functions. The ideological discourse is crafted in such a way that it casts the constructed enemy to be a ubiquitous and an existential threat. The perceived ubiquitous nature of the constructed enemy ensures that fear and paranoia dictate the actions of otherwise peaceful neighbors. Fear and paranoia are evident in hearing demands from ordinary citizens who have turned violent to know the enemy’s “plan to spread corona” or xenophobic calls to “go back to Pakistan.” In sum, once the constructed enemy becomes reified in the minds of citizens, ideologues step back and watch their ideology come to fruition; all too often without having to engage in its direct execution.

In the period of COVID-19, Hindutva no longer remains a set of racist ideas operating in the imaginations of Hindu nationalists who harbor nostalgic visions of India being a space uncontaminated by Islam. Shielded behind the veil of COVID-19, Hindutva has quickly transitioned from violent ideas to violent acts.^1^ Indeed, today it is an ideology that is materializing into violent acts, and the frequency of such acts risk making them seem increasingly mundane. Worst of all, still, is the fact that Hindutva’s violent acts are being carried out not by a paramilitary force of the BJP but rather by ordinary citizens who have accepted, as truth, fear- and paranoia-arousing propaganda from ideologues. This once again captures the discursive nature of organized ideological discourse.

## Disrupting politicized COVID-19 discourse

Using the persecution of Muslims in India that is currently taking place in the backdrop of the COVID-19 global pandemic as an illustrative case, this essay identifies the dynamics of the organization of ideological discourse by populist leaders in times of unexpected crisis. The organization of ideological discourse is engendered through a three-stage process. In the first stage, surface-level validation by legitimate institutional actors confirms preconceived ideas about a constructed enemy. In the second stage, inflammatory rhetoric is deployed by populist leaders, which scapegoat that constructed enemy. These two stages culminate to create widespread moral panic in society. With moral panic firmly established, in the third stage an environment of fear and paranoia becomes susceptible to the enactment of symbolic and physical violence against the constructed enemy.

It bears underscoring that the organization of ideological discourse by populist leaders in times of unexpected crisis is mobilized to yield their underlying political objectives and not, necessarily, to create new ones. Indeed, much akin to how Kristallnacht was not the source of antisemitism in the Nazi ideology, so too did COVID-19 not cause Islamophobia in the BJP’s Hindutva ideology. On the contrary, each of these unexpected crises were exploited by populist leaders to justify, as well as to expedite, the very religious persecution that their ideologies always desired. COVID-19, in the case of the BJP, gave its leaders the license to continue to chart the course, though now only quicker, towards the ideological project that it always possessed—a motherland based on Hindu culture and values (and without Muslims).

Given the times in which we are living, it seems apropos to close this essay with some cautionary words related to COVID-19—though these words may have just as much relevance to other unexpected crises that may emerge in the future. At present, much energy has been preoccupied with reorganizing personal and professional lives in the era of social distancing. Most individuals’ social realities have been fundamentally changed; and time and attention, understandably so, have been directed towards acclimating to the changes. Because of COVID-19, we are in the process of creating new ways of living and alternative ways of relating. Even those branches of society that are charged with tackling COVID-19 directly—involving certain members in media, healthcare, academia, industry, state bureaucracy, nongovernmental organizations—have mainly attended to the more technical aspects of the virus, answering important questions like how best to contain it or how to cope with its medical or economic effects. While undoubtedly these were (and remain) important questions, preoccupation with such issues has engendered an inadvertent consequence. It has caused a dangerous form of societal tunnel vision, preventing critical examination on how the very discourses over COVID-19 are being discretely packaged to accomplish problematic ideological aims. COVID-19 has proffered populist leaders around the world with the necessary cover to advance hateful ideologies that often call for violence against constructed enemies. Because this phenomenon has materialized in the shadows of the *politically neutral* reality of COVID-19, such ideologies have neither encountered meaningful critique nor objection.

This essay has illuminated how populist leaders of the BJP have taken full advantage of the current unexpected crisis of COVID-19 to promote their ideology of Hindutva. The anti-Muslim tenor that clandestinely foregrounds Hindutva, when it is cloaked in the concerns over the management of COVID-19, is obfuscated. Of course, it is not only countries in the Global South that are using COVID-19 to achieve political ends; as, for instance, the unsettling crackdown by Viktor Orban, the Prime Minister of Hungary, on basic democratic principles such as a free press and free and open elections has shown. Indeed, populist leaders in the Global South and the Global North adopt a very similar playbook to ascertain and maintain power (Masood and Nisar, 2020). For this reason, it would not take too great a leap of faith to imagine scenarios in which, for example, Donald Trump invokes COVID-19 in attempts to suspend the presidential election slated for later this year or, otherwise, has members of his republican party use the pandemic to further engage in acts of voter suppression in efforts to guarantee his second term in office.

The organization of ideological discourse is part of the intricate machinery invoked by populists to craft social realities that are aligned to their own political and ideological aspirations. Citizens have the responsibility to decipher between truth and false, real and imagined, and fact and fiction (Prasad, 2019). As the effects of COVID-19 unfolds, it is crucial for citizens to be wary of the discourses about the disease coming from political leaders. Leaders who make truth-claims that are not substantiated by evidence-based science ought to be received with suspicion—repudiated when necessary. While the effects of COVID-19 are very much real—at the time of writing, 3.6 million individuals have been infected with the virus and more than 250,000 lives have succumbed to it—some of what political leaders have had to say about the disease is questionable, distorted, or purposefully deceptive. If we are to take seriously George Santayana’s words that I quoted to introduce this essay, we ought to keep remembrance of the lessons from the Night of the Broken Glass as we try to navigate this truly global pandemic.

## Note

1 This assertion is not to suggest the Hindutva has not previously been associated with violent acts. Indeed, Hindutva has a long history of both directly and indirectly engendering violence against non-Hindus, and especially Muslims. A case in point was the 2002 communal riots in the state of Gujarat, which claimed the lives of over 1000 people. The communal riots occurred during a time when Modi was the Chief Minister of the state. Given Modi’s—and the BJP’s—adherence to Hindutva, it is not surprising that he, at the very least, tacitly sanctioned the communal violence against Muslims (Masood and Nisar, 2020). So, while the ideology of Hindutva is ontologically violent, the point being offered here is that COVID-19 has allowed for ideologues to issue incendiary statements that have only intensified violence against Muslims in terms of degree and frequency.
